# An Incremental Broad-Learning-System-Based Approach for Tremor Attenuation for Robot Tele-Operation

**DOI:** 10.3390/e25070999

**Published:** 2023-06-29

**Authors:** Guanyu Lai, Weizhen Liu, Weijun Yang, Huihui Zhong, Yutao He, Yun Zhang

**Affiliations:** 1 School of Automation, Guangdong University of Technology, Guangzhou 510006, China; lgy124@foxmail.com (G.L.); lwz0906@foxmail.com (W.L.); 3221001032@gdut.edu.cn (H.Z.); heyutao81@foxmail.com (Y.H.); yun@gdut.edu.cn (Y.Z.); 2 School of Mechanical and Electrical Engineering, Guangzhou City Polytechnic, Guangzhou 510405, China

**Keywords:** hand physiological tremors, incremental broad learning system, tele-operation robot system, sliding mode controller

## Abstract

The existence of the physiological tremor of the human hand significantly affects the application of tele-operation systems in performing high-precision tasks, such as tele-surgery, and currently, the process of effectively eliminating the physiological tremor has been an important yet challenging research topic in the tele-operation robot field. Some scholars propose using deep learning algorithms to solve this problem, but a large number of hyperparameters lead to a slow training speed. Later, the support-vector-machine-based methods have been applied to solve the problem, thereby effectively canceling tremors. However, these methods may lose the prediction accuracy, because learning energy cannot be accurately assigned. Therefore, in this paper, we propose a broad-learning-system-based tremor filter, which integrates a series of incremental learning algorithms to achieve fast remodeling and reach the desired performance. Note that the broad-learning-system-based filter has a fast learning rate while ensuring the accuracy due to its simple and novel network structure. Unlike other algorithms, it uses incremental learning algorithms to constantly update network parameters during training, and it stops learning when the error converges to zero. By focusing on the control performance of the slave robot, a sliding mode control approach has been used to improve the performance of closed-loop systems. In simulation experiments, the results demonstrated the feasibility of our proposed method.

## 1. Introduction

With the rapid advancements of tele-operation techniques, robots have gradually improved in performance and have been applied for various areas such as medical and space exploration, see [1,2,3] for examples, where they are used to complete difficult and complicated scenes with greater precision and efficiency. The stability of tele-operating systems is susceptible to various factors, such as human hand tremors and transmission time delays. Hand tremors in tele-operation lead to suboptimal task tracking. The physiological tremors in human hands are natural, and not pathological [4,5]. These tremors exist in every part of the human body with an amplitude range between 50 and 100 μm in each principal axis, and their dominant frequency is usually distributed in the range of 8–12 Hz [6,7]. Note that physiological tremors are intolerant in the tele-operation scene requiring highly precise manual positioning [8,9,10], since they can make a remote robot generate motion deviations. Hence, it is imperative to compensate for these tremor signals to enhance the effectiveness of tele-robotic operation systems. To eliminate this influence of tremors, various related methods have been proposed; see [11,12,13,14,15,16,17] for examples.

Since physiological tremors exhibit a high-frequency characteristic, while human hand motion is low frequency, some scholars have proposed utilizing the linear low-pass filter [11], which can filter out high-frequency signals and retain low-frequency signals. However, digital filters usually require caching and data processing, which can cause time delays and affect the response speed of systems. The literature show the results of the implementation of canceling tremors on tele-operation systems using the low-pass filter, thereby demonstrating that it is fundamental to set the filter frequency threshold, wherein the optimal frequency threshold still loses some information [12]. To address the limitations of digital filters, in [13], T. A. Wei and P. K. Khosla proposed that the Kalman filter (KF) can combine sensor measurements and dynamic system modeling, as well as estimate the state of the system using the Kalman filter principle, to eliminate the tremor. Furthermore, in [14], Y. Wang proposed an innovative band-limited multiple Fourier linear combiner (BMFLC)-based KF approach (BMFLC-KF) to offer the decomposition of band-limited signals in the time frequency, thereby facilitating effective filtering and compensation. In [15], the authors proposed an autoregressive-based KF model (AR-KF) aimed at the real-time estimation of oscillatory patterns by leveraging past output data. In [16], the authors proposed algorithms based on multi-step (MS) prediction to address the phase delays in the sensors and filters, and they accurately eliminated real-time tremors. Since the fusion of various algorithms leads to computational cost increases, a reduced-order Kalman-enhanced-based BMFLC model (RKE-BMFLC) was proposed in [17], which can reduce the computational complexity of the system and improve real-time performance. Despite their promising performance in predicting tremors, the algorithms mentioned above still have certain limitations. First, the AR-KF method utilizes a linear prediction model to represent the tremor signal as a linear Gaussian distribution. To achieve accurate results, the KF approach must consider the specific characteristics of the tremor being analyzed. Second, when applying the BMFLC-KF, one may need to select parameters carefully, and as shown in [14]; the results clearly show that a minor frequency gap can lead to the infeasibility of such an approach for accurate estimation.

To remove the two limitations above, many machine learning-based approaches have been proposed, e.g., the small-scale sample learning method has been employed widely in physiological tremor elimination applications for tele-operation systems. In [18], Luo, J. proposed the support vector machine (SVM) algorithm as the model for tremor cancellation, wherein it demonstrated good generalization ability and excellent computational performance. In [19], Z. Liu made the SVM algorithm more adaptable to remote operating system tasks and proposed an adaptive fuzzy SVM-based algorithm filter, which filters time series signals and is capable of more accurately modeling tremor signals. In addition to the small-scale sample approach, strong deep learning models [20,21,22] can further learn the characteristics of tremor signals and achieve high-precision tremor elimination. Despite the merits of the machine-learning-based approaches such as those mentioned above, they still have some restrictions: (1) for a small-scale sample learning method, a loss in prediction accuracy may occur, because learning energy cannot be accurately assigned, and it may be insensitive to small amplitude signals, which may result in poor performance; (2) for deep learning models, a large number of hyperparameters is an unavoidable problem, regardless of their ability to process data efficiently.

Motivated by the observation above, in this study, an incremental broad learning system filter (I-BLSF) has been proposed to predict and cancel hand physiological tremors. In summary, the main novelties and contributions of this work are listed as follows:

- Unlike high-complexity deep learning networks, a simple and efficient network, broad learning system (BLS), is applied in tele-operation systems as a tremor filter, which overcomes the shortcomings of traditional deep neural networks by using the pseudo-inverse calculation. Due to the ill-posed problem, we combine the BLS with the ridge regression approach.
- Traditional batch-learning algorithms require a lot of time and computing resources, and they are limited in dealing with mass data. To solve the problem, incremental learning algorithms are introduced to rebuild the network model online, which can improve the model performance.
- A novel sliding mode controller is raised. The previous work [23] combined with the PD controller to achieve tremor canceling, and there was still room for improvement in tracking accuracy and robustness. Thus, in this paper, we apply a superior controller to control the slave robot.

The rest of this paper is structured as follows. First, Section 2 points out the research problem that needs to be addressed. Then, Section 3 describes the control strategies designed for teleoperation. The proposed broad-learning-system-based filter (BLSF) approach is introduced in Section 4. Section 5 shows experiment parameters and Section 6 validates by simulation that our proposed method has the capability for canceling tremors. Finally, Section 7 makes a summary of the paper.

## 2. Problem Description

The background problem of this study will be described in this section. First, we introduce the tele-operation system, including the master and slave devices. Then, in the master part, the joints of the master device are analyzed. Finally, the workspace relationship between the master and the slave is given.

### 2.1. Tele-Operated Robot System

**Figure 1:**
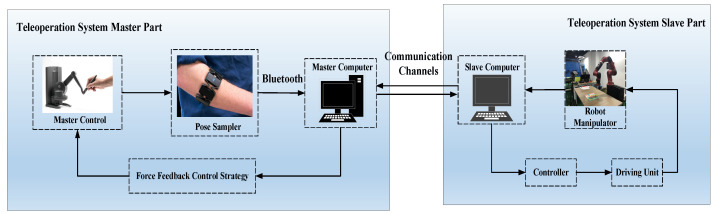
Tele-Operation robot system elements.

In Figure 1, it shows components of a tele-operation robot system, which is composed of the following parts: (1) the master part (involving a haptic device and a sampling device); (2) the bluetooth communication channel; and (3) the slave part (containing a slave robot manipulator).

- **Haptic device and sampling device:** The haptic device contains a six degrees of freedom (DOFs), where the first three are used to describe the position of the haptic device, and the last three are used to describe the orientation of the haptic device. The sampling device (Myo armband) has eight electromyography (EMG) electrodes and one nine-axis inertial measurement unit (IMU), which can obtain the change in human arm muscle bioelectricity versus time.
- **Communication channels:** Bluetooth technology eliminates the need for wires between master devices and slave devices through wireless connections. Master–slave computers can communicate with each other at a certain distance through a wireless receiver on the chip.
- **Slave robot manipulator:** A multi-DOFs robot manipulator is used as the slave control object, which is equipped with force sensors and electric servers on each joint, where electric servers include the control circuit, direct current (DC) motor, and reduction gear set.

### 2.2. Master Joints Analysis

Although physiological tremors are normal signals in our daily life, they are a non-negligible issue for meeting about 10 μm range position accuracy [24]. These tremor signals affect each joint of the master device by yielding disturbance signals. In this paper, the modified D-H notation [25] has been adopted to express a haptic device with tremors, and we have the following equation:

(1) $$\begin{array}{c} \left\{\begin{array}{cc} \;\theta_{ori_{i}}+\theta_{h_{i}}+\Delta \theta_{i}=\theta_{new_{i}},\;i=1,2,3,4,5,6, & \\ \;d_{ori_{i}}+d_{h_{i}}+\Delta d_{i}=d_{new_{i}},\;i=1,2,3,4,5,6, \end{array}\right. \end{array}$$

where $\theta_{ori}$ and $d_{ori}$ are the original joint information, $\theta_{h}$ and $d_{h}$ are the desired values from the human hand, and *i* represents the *i*-th joint. Δθ and Δd are the disturbed values influenced by tremors, and $\theta_{new}$ and $d_{new}$ are actual joint information. And then, the homogeneous transformation matrix can be described in the following form:

(2) $$\begin{array}{cc} {}_{i-1}^{i}\hat{T}=\left[\begin{array}{cccc} c\theta_{new_{i}} & -s\theta_{new_{i}} & 0 & a_{i-1}\\ s\theta_{new_{i}}c\alpha_{i-1} & c\theta_{new_{i}}c\alpha_{i-1} & -s\alpha_{i-1} & -s\alpha_{i-1}sd_{new_{i}}\\ s\theta_{new_{i}}s\alpha_{i-1} & s\alpha_{i-1}c\theta_{new_{i}} & c\alpha_{i-1} & c\alpha_{i-1}d_{new_{i}}\\ 0 & 0 & 0 & 1 \end{array}\right] & ,\\ s=sin(\cdot ),c=cos(\cdot ),i=2,\ldots ,6, \end{array}$$

where α is the kinematic link twist. To obtain the joint transformation matrix for the end effector, the six matrixes ${}_{1}^{0}\hat{T}$, ${}_{2}^{1}\hat{T}$, ${}_{3}^{2}\hat{T}$, ${}_{4}^{3}\hat{T}$, ${}_{5}^{4}\hat{T}$, and ${}_{6}^{5}\hat{T}$ can be multiplied in a sequence as follows:

(3) $${}_{6}^{0}\hat{T}={\;}_{1}^{0}\hat{T}{\;}_{2}^{1}\hat{T}{\;}_{3}^{2}\hat{T}{\;}_{4}^{3}\hat{T}{\;}_{5}^{4}\hat{T}{\;}_{6}^{5}\hat{T}=\left[\begin{array}{cccc} {\hat{n}}_{11} & {\hat{n}}_{12} & {\hat{n}}_{13} & {\hat{p}}_{x}\\ {\hat{n}}_{21} & {\hat{n}}_{22} & {\hat{n}}_{23} & {\hat{p}}_{y}\\ {\hat{n}}_{31} & {\hat{n}}_{32} & {\hat{n}}_{33} & {\hat{p}}_{z}\\ 0 & 0 & 0 & 1 \end{array}\right],$$

where ${\hat{n}}_{ij},i=1,2,3,j=1,2,3$, and ${\hat{p}}_{x}$, ${\hat{p}}_{y}$, ${\hat{p}}_{z}$ are the rotational factors and the position vector, respectively.

### 2.3. Workspace Description

In a tele-opeation robot system, the description of the coordinate system between the master and the slave is different due to their different physical characteristics, and their workspace relationship is shown as follows:

(4) $$S_{s}=Z_{\delta }\times \left(\theta S_{m}+b\right),$$

where $S_{s}$ defines the coordinates of the slave robot manipulator, and $S_{m}$ defines the coordinates of the master device. $Z_{\delta }$ is the rotational matrix about the *z*-axis, and Equation (4) has the following form:

(5) $$\begin{array}{cc} \left[\begin{array}{c} x_{s}\\ y_{s}\\ z_{s} \end{array}\right]= & \left[\begin{array}{ccc} cos\delta & -sin\delta & 0\\ sin\delta & cos\delta & 0\\ 0 & 0 & 1 \end{array}\right]\times \left(\left[\begin{array}{ccc} \theta_{x} & 0 & 0\\ 0 & \theta_{y} & 0\\ 0 & 0 & \theta_{z} \end{array}\right]\left[\begin{array}{c} x_{m}\\ y_{m}\\ z_{m} \end{array}\right]+\left[\begin{array}{c} b_{x}\\ b_{y}\\ b_{z} \end{array}\right]\right), \end{array}$$

where δ is a rotation angle. $\theta_{x}$, $\theta_{y}$, and $\theta_{z}$ are the scale factors in the three-axis direction, and $b_{x}$, $b_{y}$, and $b_{z}$ are the translation factors the three-axis direction. The parameters of Equation (5) are provided as below:

(6) $$\begin{array}{c} \left\{\begin{array}{cc} \delta =\frac{\pi }{4} & \\ {\left[\theta_{x}\;\theta_{y}\;\theta_{z}\right]}^{T}={\left[0.041\;0.040\;0.041\right]}^{T} & \\ {\left[b_{x}\;b_{y}\;b_{z}\right]}^{T}={\left[0.701\;0.210\;0.129\right]}^{T}. & \end{array}\right. \end{array}$$

The presence of tremors in the homogeneous transformation matrix of a haptic device leads to changes in the master coordinates $X_{m}$, which in turn causes differences in the slave coordinates $X_{s}$. As the error in the master–slave position increases, the accuracy of the system decreases. To address this issue, a tremor attenuation filter can be designed to reduce the effects of tremors on the performance of tele-operation systems.

## 3. Control Strategies

**Figure 2:**
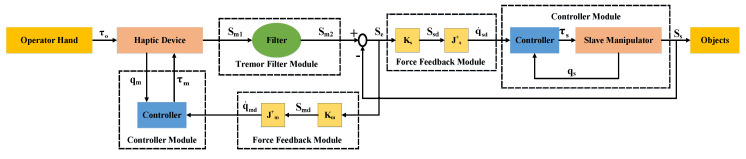
Control mode in teleoperation systems.

In the paper, we integrated the force feedback module, the controller module, and the tremor filter module into the teleoperation system. An initial torque $\tau_{o}$ from a human hand is sent to the haptic device, and the haptic sends an operation trajectory $S_{m1}$ to the filer unit. $S_{m2}$ is a filtered trajectory, $S_{s}$ is an actual trajectory of the slave manipulator, and $S_{e}$ is an error trajectory, i.e., $S_{e}=S_{m2}-S_{s}$. The error signal is sent to the force feedback module to obtain the master and slave control variables ${\dot{q}}_{md}$ and ${\dot{q}}_{sd}$, respectively. In the controller module, the controller exports the master and slave torque, which are $\tau_{m}$ and $\tau_{s}$, respectively. Figure 2 shows this process in tele-operation robot systems. Here, we provide more details on the control strategies as follows.

### 3.1. Force Feedback Control

When the end effector of the slave robot arm follows the motion of the master device, the master device can receive feedback information from the force sensors of the slave robot joints. Force feedback can achieve the integration of visual perception and tactile sensation, thus ensuring that the operator can perceive the remote environment and manipulate the robot more naturally [26]. The strength of the feedback force is expressed as [27]:

(7) $$F_{f}=K_{f}\sqrt{{\left(x_{s}-x_{m}\right)}^{2}+{\left(y_{s}-y_{m}\right)}^{2}+{\left(z_{s}-z_{m}\right)}^{2}},$$

where $x_{m}$, $y_{m}$, and $z_{m}$ and $x_{s}$, $y_{s}$, and $z_{s}$ are the coordinate values of the master device and the slave device, respectively. $K_{f}$ is a feedback force parameter. With the optimization of a received feedback force from the slave part, the desired trajectories of the master and slave devices, $S_{md}$ and $S_{sd}$, respectively, can be obtained. The pose information can be turned into the joint velocity information by the Jacobian matrixes $J_{m}^{+}$ and $J_{s}^{+}$ as follows:

(8) $$\begin{array}{c} \left\{\begin{array}{cc} {\dot{q}}_{md}=J_{m}^{+}\left(\dot{S_{m}}\right){\dot{S}}_{md}, & \\ {\dot{q}}_{sd}=J_{s}^{+}\left(\dot{S_{s}}\right){\dot{S}}_{sd}. & \end{array}\right. \end{array}$$

### 3.2. Sliding Mode Controller

The sliding mode controller, known for its ability to overcome system uncertainties and achieve robust control characteristics [28], employs different structures on both sides of the sliding surface. This nonlinear controller is particularly effective in dealing with the complexities of uncertain dynamic systems. For a typical second-order nonlinear uncertain dynamic system with a single input, its general state space expression can be described as follows [29]:

(9) $$\dot{x_{1}}\left(t\right)=x_{2}\left(t\right)$$

(10) $$\dot{x_{2}}\left(t\right)=\sum_{i=1}^{n}\left(a_{i}+\Delta_{i}\left(t\right)\right)f_{i}\left(x_{1},x_{2},t\right)+b\left(x_{1},x_{2},t\right)u\left(t\right)+d\left(t\right)$$

(11) $$x_{1}\left(t_{0}\right)=x_{1,0},x_{2}\left(t_{0}\right)=x_{2,0},$$

where $x_{1}\left(t\right)$ and $x_{2}\left(t\right)$ are the state variables, $a_{i}\;\left(i=1,\cdots ,n\right)$ are the constant parameters of the system, and $\Delta_{i}$ and d(t) are the uncertain perturbations and the known disturbance, respectively. u(t) represents the input signals, and $f_{i}\left(x_{1},x_{2},t\right)$ and $b(x_{1},x_{2},t)$ are derived by the system characteristics; $x_{1,0}$ and $x_{2,0}$ are initial conditions given at the initial time $t_{0}$. The main objective of this controller is to satisfy that $X\left(t\right)={\left[x_{1}\left(t\right),x_{2}\left(t\right)\right]}^{T}$ can track the desired trajectory $X_{d}\left(t\right)=\left[x_{d1}\left(t\right),x_{d2}\left(t\right)\right]$. Hence, the control law should be designed to make the tracking error asymptotically arrive at zero. Since the above considered system is single input, there exists only one sliding surface $s(x_{1},x_{2})=0$ for second order systems, and it is defined as follows:

(12) $$s\left(x_{1},x_{2}\right)=err_{2}\left(t\right)+c\times err_{1}\left(t\right),$$

where *c* is a strictly positive real number, and the tracking errors $err_{1}\left(t\right)$ and $err_{2}\left(t\right)$ are written as follows:

(13) $$\begin{array}{cc} \; & err_{1}\left(t\right)=x_{1}\left(t\right)-x_{d1}\left(t\right)\\ \; & err_{2}\left(t\right)=x_{2}\left(t\right)-x_{d2}\left(t\right). \end{array}$$

Assume that ${\dot{err}}_{1}=err_{2}$, and denote $E\left(t\right)=[err_{1}\left(t\right),err_{2}\left(t\right)]$. To obtain a unique solution of a homogeneous differential equation err(t)=0, $s(x_{1},x_{2})$ is set as zero. Thus, the tracking error will asymptotically reach zero with a proper control law that can keep the trajectory on the sliding surface. The control law is designed as follows:

(14) $$\begin{array}{cc} \; & \dot{s}={\dot{err}}_{2}+c\times err_{2}=-bsgn\left(s\right),b>0\\ \; & {\dot{err}}_{2}=-c\times err_{2}-bsgn\left(s\right),b>0, \end{array}$$

where *b* is a positive number.

**Remark 1.**  *Traditional controllers often rely on control algorithms such as the PID controller, which are simple and easy to implement but have limited accuracy and anti-interference capabilities. In contrast, the sliding mode control is effective in reducing the effects of uncertainties and external disturbances that are common in practical systems, which is achieved by designing a sliding surface that drives the system towards a stable equilibrium point, regardless of the uncertainties and disturbances. Furthermore, the sliding mode control provides a fast response and high tracking accuracy.*

### 3.3. Tremor Attenuation Filter

**Figure 3:**
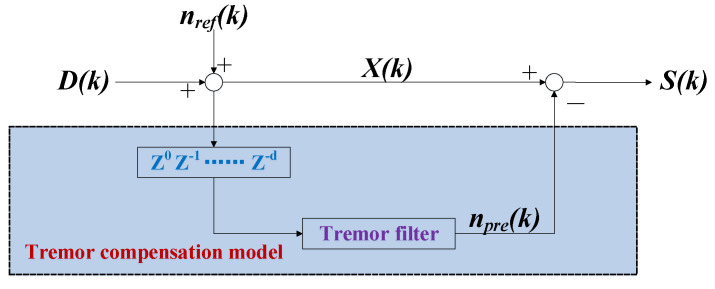
Mathematical expression of the tremor complementation model.

To provide a more detailed explanation of the flow of signals in the tremor filter, we provide the mathematical model of the designed tremor filter in Figure 3. The model illustrates the various flows involved in the filtering process and how they interact with each other.

Since the data sampled by the sampling unit are hand trajectories and tremor disturbance signals, we denote the input of the tremor filter mathematical model as actual signals X(k) and the tremor disturbance signals as $n_{ref}\left(k\right)$. The actual signals with tremors can be written as follows:

(15) $$X\left(k\right)=D\left(k\right)+n_{ref}\left(k\right),$$

where *k* is the sampling point, and D(k) is the desired signal without tremors. Through the prediction of the tremor filter, the output of the tremor filter mathematical model is

(16) $$S\left(k\right)=X\left(k\right)-n_{pre}\left(k\right)=D\left(k\right)+n_{ref}\left(k\right)-n_{pre}\left(k\right),$$

where $n_{pre}\left(k\right)$ is the prediction signal of the tremor filter.

We denote the error as $\Delta n=n_{ref}\left(k\right)-n_{pre}\left(k\right)$ and aim for it to equal to zero. Theoretical predictions suggest that the model error ideally should be zero. In practical scenarios, there might exist a small residual deviation, thus resulting in a prediction error that is slightly larger than zero.

## 4. Design of Broad-Learning-System-Based Tremor Filter

While deep learning algorithms are efficient at processing large amounts of data, they often involve a large number of hyperparameters, which can be problematic. The broad learning system is a novel and efficient network architecture that avoids the complex and redundant structures found in traditional deep learning networks [30,31]. As a result, it provides a more efficient, interpretable, and scalable solution for processing data.

### 4.1. Broad Learning System

**Figure 4:**
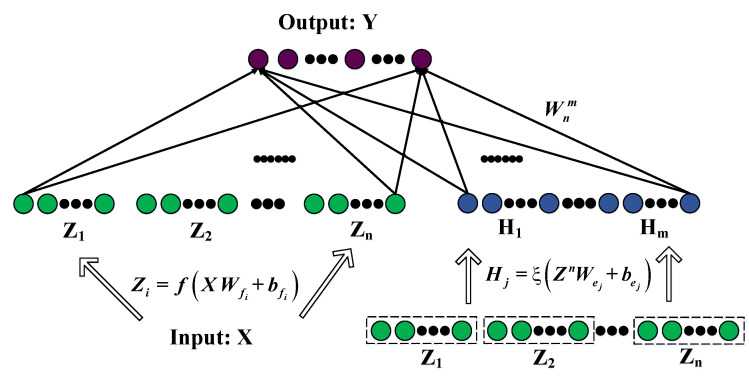
Broad learning system network model architecture.

The proposed network architecture was developed by C. L. Philip Chen and is referred to as the broad learning system, which is depicted in Figure 4. This novel network architecture differs from deep learning neural networks, as it does not require backpropagation to update weights. The speed of the broad learning system is attributed to the fact that weights can be obtained via pseudo-inverse formulas. Moreover, the network weights are continuously updated as the system is trained with data, as the system employs an incremental learning algorithm to adjust nodes without reinputting previous data.

In Figure 4, we denote $X\in R^{M\times N}$ as the input into the BLS, and we denote $Y\in R^{M\times C}$ as the output, where *M*, *N*, and C represent the number of samples, the number of features, and the number of output nodes, respectively. The input data *X* is randomly mapped to *n* sets of feature window nodes, thereby generating the feature layer of the network, which can be expressed in the following form:

(17) $$Z_{i}=\phi \left(XW_{f_{i}}+\beta_{f_{i}}\right),\;i=1,\ldots ,n,$$

where the variables $W_{f_{i}}$ and $\beta_{f_{i}}$ correspond to randomly generated weights and biases, respectively. ϕ(·) refers to a random mapping function. Each mapping group contains *k* feature nodes. All the feature nodes can be represented as $Z^{n}\equiv \left[Z_{1},Z_{2},\cdots ,Z_{n}\right]$, and we denote *j*-th group enhancement nodes as the following:

(18) $$H_{j}=\xi \left(\left[Z_{1},Z_{2},\cdots ,Z_{n}\right]W_{hj}+\beta_{hj}\right)=\xi \left(Z^{n}W_{e_{j}}+\beta_{e_{j}}\right),\;j=1,...,m,$$

where $W_{hj}$ and $\beta_{hj}$ are random weight coefficients, and the function ξ(·) is a nonlinear activation function, f=tanh(·). We denote all nodes as $L_{n}^{m}\equiv \left[Z_{1},Z_{2},\cdots ,Z_{n}|H_{1},H_{2},\cdots ,H_{m}\right]$. The output of the broad learning model is represented as follows:

(19) $$\begin{array}{cc} Y & =\left[Z_{1},\cdots ,Z_{n}|\xi \left(Z^{n}W_{h1}+\beta_{h1}\right),\cdots ,\xi \left(Z^{n}W_{hm}+\beta_{hm}\right)\right]W_{n}^{m}\\ \; & =\left[Z_{1},\cdots ,Z_{n}|H_{1},\cdots ,H_{m}\right]W_{n}^{m}=L_{n}^{m}W_{n}^{m}. \end{array}$$

Here, $W_{n}^{m}$ represents the connection weight of the network with *n* feature windows and *m* groups of enhancement nodes. For all nodes $L_{n}^{m}$, the pseudoinverse is equal to the following:

(20) $${\left(L_{n}^{m}\right)}^{+}={\left[{\left(L_{n}^{m}\right)}^{T}L_{n}^{m}\right]}^{-1}{\left(L_{n}^{m}\right)}^{T},$$

and the weights can be represented as follows:

(21) $$W_{n}^{m}={\left(L_{n}^{m}\right)}^{+}Y={\left[{\left(L_{n}^{m}\right)}^{T}L_{n}^{m}\right]}^{-1}{\left(L_{n}^{m}\right)}^{T}Y.$$

Due to the ill-posed nature of the problem, where no stable or unique solution to the inverse matrix exists, the ridge regression algorithm is employed to obtain the connection weights of the structure. As a result, Equation (20) can be rewritten as follows:

(22) $${\left(L_{n}^{m}\right)}^{+}=\lim_{\lambda \to 0}\left({\left[\lambda E+{\left(L_{n}^{m}\right)}^{T}L_{n}^{m}\right]}^{-1}{\left(L_{n}^{m}\right)}^{T}\right),$$

and we have

(23) $$W_{n}^{m}={\left(L_{n}^{m}\right)}^{+}Y=\lim_{\lambda \to 0}\left({\left[\lambda E+{\left(L_{n}^{m}\right)}^{T}L_{n}^{m}\right]}^{-1}{\left(L_{n}^{m}\right)}^{T}\right)Y.$$

**Remark 2.**  *The broad learning model (BLM) is a computational framework that offers a fast and efficient solution for various supervised and unsupervised machine learning tasks. The BLM has been developed to overcome the limitations of traditional deep learning architectures, which typically require a large number of layers and a large amount of computational resources to achieve high predictive performance. The singular value decomposition technique is used to simplify the complexity of the model, and incremental learning modes can be integrated to form the broad learning system.*

### 4.2. Incremental Learning Methods

In the broad learning system, to improve the system performance, an incremental learning approach is integrated. This incremental learning method has three updating forms, which contain the increment of the feature nodes, the increment of the enhancement nodes, and the increment of the input data. Since the input data is enough for our experiment, in this paper, the first two methods are considered. The details are given as follows.

#### 4.2.1. Increment of Additional Enhancement Nodes

Denote $L_{n}^{m}=\left[\;Z_{n}\;|\;H_{m}\;\right]$ and denote the group of additional enhancement nodes as $H^{*}=\xi \left(Z^{n}W_{h(m+1)}+\beta_{h(m+1)}\right)$. Hence, the new input matrix is written as follows:

(24) $$\begin{array}{c} L^{m+1}\equiv \left[\;L_{n}^{m}\;|\;\xi \left(Z^{n}W_{h(m+1)}+\beta_{h(m+1)}\right)\;\right], \end{array}$$

where $W_{h(m+1)}$ and $\beta_{h(m+1)}$ are random weights and random biases from n groups of features mapping to p additional enhancement nodes, respectively. The pseudo-inverse of the new matrix can be written as follows:

(25) $${\left(L^{m+1}\right)}^{+}=\left[\begin{array}{c} {\left(L_{n}^{m}\right)}^{+}-DB^{T}\\ B^{T} \end{array}\right],$$

where $D={\left(L_{n}^{m}\right)}^{+}\xi \left(Z^{n}W_{h(m+1)}+\beta_{h(m+1)}\right)$,

(26) $$B^{T}=\left\{\begin{array}{cc} {\left(C\right)}^{+} & if\;C\neq 0\\ {\left(1+D^{T}D\right)}^{-1}D^{T}{\left(A_{n}^{m}\right)}^{+} & if\;C=0, \end{array}\right.$$

and $C=\xi \left(Z^{n}W_{h(m+1)}+\beta_{h(m+1)}\right)-A_{n}^{m}D$; the new weights are denoted as the following:

(27) $$W^{m+1}=\left[\begin{array}{c} W^{m}-DB^{T}Y\\ B^{T}Y \end{array}\right].$$

**Remark 3.**  *When the trained network fails to achieve the desired accuracy, additional enhancement nodes can be added to improve the accuracy. By adding extra enhancement nodes into the network, the nonlinear capability can be enhanced. As shown in the equations above, the algorithm only requires the calculation of the pseudo-inverse of the new nodes rather than the entire matrix, thereby enabling the network to be rapidly restructured.*

#### 4.2.2. Increment of Additional Feature Mapping Nodes

We point out that the dynamic increment of the enhancement nodes method cannot improve the current network performance, as it may fall into a locally optimal solution. The increment of additional feature mapping nodes is an effective learning method for neural networks, which only needs to calculate the pseudo-inverse of the new nodes and does not need to retrain the whole network. This method provides the benefits of saving time for improving the feature extraction capability.

Assume that the initial nodes are constructed by n groups of feature mapping nodes and m groups of enhancement nodes, and denote the additional (*n*+1)-th group feature mapping nodes as $Z_{n+1}=\phi \left(XW_{e(n+1)}+\beta_{e(n+1)}\right)$, where $W_{e(n+1)}$ and $\beta_{e(n+1)}$ are randomly generated. The corresponding enhancement nodes generated by the additional (n+1)-th group feature mapping nodes are defined as follows:

(28) $$H_{j}^{*}=\left[\xi \left(Z_{n+1}W_{e1}^{*}+\beta_{e1}^{*}\right),\xi \left(Z_{n+1}W_{e2}^{*}+\beta_{e2}^{*}\right),\cdots ,\xi \left(Z_{n+1}W_{ej}^{*}+\beta_{ej}^{*}\right)\right],\;j=1,...,m,$$

where $W_{ei}^{*}$ and $\beta_{ei}^{*}$ are random parameters. Here, we denote $L_{n+1}^{m}=[\;L_{n}^{m}\;|\;Z_{n+1}\;\left|\;H_{j}^{*}\;\right]$, and its pseudo-inverse matrix is defined as follows:

(29) $${\left(L_{n+1}^{m}\right)}^{+}=\left[\begin{array}{c} {\left(L_{n}^{m}\right)}^{+}-db^{T}\\ b^{T} \end{array}\right],$$

where $d={\left(L_{n+1}^{m}\right)}^{+}\left[\;Z_{n+1}\;|\;H_{j}^{*}\;\right]$,

(30) $$b^{T}=\left\{\begin{array}{cc} {\left(c\right)}^{+} & if\;c\neq 0\\ {\left(1+d^{T}d\right)}^{-1}d^{T}{\left(L_{n}^{m}\right)}^{+} & if\;c=0, \end{array}\right.$$

and $c=\left[\;Z_{n+1}\;|\;H_{j}^{*}\;\right]-L_{n}^{m}d$; the new weights are denoted as follows:

(31) $$W_{n+1}^{m}=\left[\begin{array}{c} W_{n}^{m}-db^{T}Y\\ b^{T}Y \end{array}\right].$$

### 4.3. Sparse Autoencoder

Obtaining a good feature representation of input data is a critical step in machine learning. Traditionally, complex mathematical derivations have been used to derive features, or a set of features has been generated through random initialization. However, random features suffer from unpredictability and uncertainty, which may lead to incomplete feature extraction. As the dimensionality or size of the input data increases, it becomes necessary to remove redundant features.

**Figure 5:**
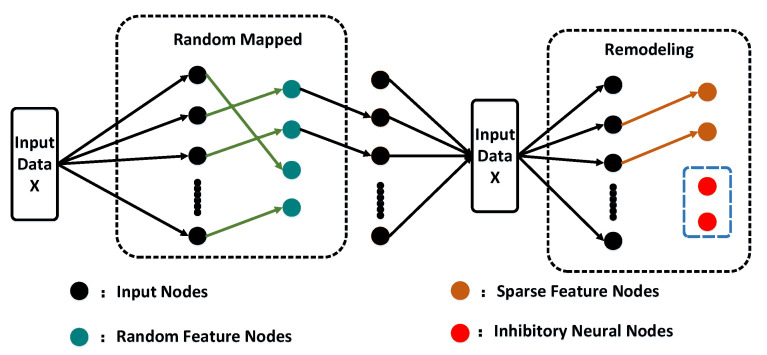
Sparse autoencoder structure diagram.

To address these issues, the sparse autoencoder (SAE) model has been proposed, which fine-tunes random features into a set of sparse and compact features [32,33,34]. The SAE model structure is illustrated in Figure 5, and then the details of the sparse feature learning algorithm are described below.

The extraction of sparse features is considered to be an optimization problem that requires addressing. Lasso regression ($l_{1}$ regularization) represents a convex optimization problem, as stated in [35]. To obtain the solution $W^{*}$ for the sparse autoencoder, the following optimization problem can be used to obtain it:

(32) $$f\left(W^{*}\right)=\underset{W^{*}}{arg min}\;∥ZW^{*}{-X∥}_{2}^{2}+C{∥W^{*}∥}_{1},$$

where *C* is the regularization parameter, and *Z* is the output of the linear random mapping equation, as shown in Equation (17). It is well-known that $l_{1}$ regularization is often used to solve linear inverse problems. A common approach is the alternating direction method of multipliers (ADMM), which is used to obtain the solution by minimizing one function at a time. To apply the ADMM algorithm, we first reformulate Equation (32) as follows:

(33) $$\begin{array}{cc} f\left(W^{*}\right)=f\left(w,v\right)= & \underset{w,v}{arg min}\;h\left(w\right)+g\left(v\right)\\ s.t\; & w-v=0, \end{array}$$

where $h\left(w\right)={∥Zw-X∥}_{2}^{2}$, $g\left(v\right)={c∥v∥}_{1}$. In the augmented Lagrangian with a penalty form, we have the following:

(34) $$\begin{array}{cc} \underset{w,v}{arg min}\; & h\left(w\right)+g\left(v\right)+\lambda \left(w-v\right)+\frac{\rho }{2}{∥w-v∥}_{2}^{2}\\ \; & s.t\;w-v=0, \end{array}$$

and then the solution of the original problem can be obtained as follows:

(35) $$\begin{array}{c} \begin{array}{cc} w_{k+1} & ={\left(Z^{T}Z+\frac{\rho }{2}I\right)}^{-1}\left[Z^{T}X+\frac{\rho }{2}\left(v_{k}-u_{k}\right)\right],\\ v_{k+1} & =S_{k}\left(w_{k+1}+u_{k}\right),\;\;k=\frac{c}{\rho },\\ u_{k+1} & =u_{k}+\left(w_{k+1}-v_{k+1}\right),\;u_{k}=\frac{\lambda^{T}}{\rho }, \end{array} \end{array}$$

where ρ is a positive penalty factor. S(·) is the soft thresholding operator, and it is defined as follows:

(36) $$\begin{array}{c} S_{k}\left(w_{k+1}+u_{k}\right)=\left\{\begin{array}{cc} w_{k+1}+u_{k}-k & if(w_{k+1}+u_{k}>k)\\ 0 & if(|w_{k+1}+u_{k}|⩽k)\\ w_{k+1}+u_{k}+k & if(w_{k+1}+u_{k}<-k) \end{array}\right.. \end{array}$$

### 4.4. Physical Model Structure of BLSF

A novel BLSF was proposed to address the effects of physiological tremors, and it consists of three main components: the sampling unit, the tremor filter unit, and the control unit. In the sampling unit, an internal measurement unit (IMU) captures real-time hand movements by measuring the three-axis position acceleration $\ddot{x}$, $\ddot{y}$, and $\ddot{z}$ and the three-axis joint angular velocity ${\dot{\theta }}_{x}$, ${\dot{\theta }}_{y}$, and ${\dot{\theta }}_{z}$. The tremor filtering unit utilizes the BLS network algorithm to forecast and compensate for tremor signals, thereby effectively neutralizing them in the actual signals. The control unit incorporates inverse kinematics calculations, single joint drivers, and motion feedback from deflection sensors to convert inverse kinematics into motion control variables for the robot manipulator.

**Figure 6:**
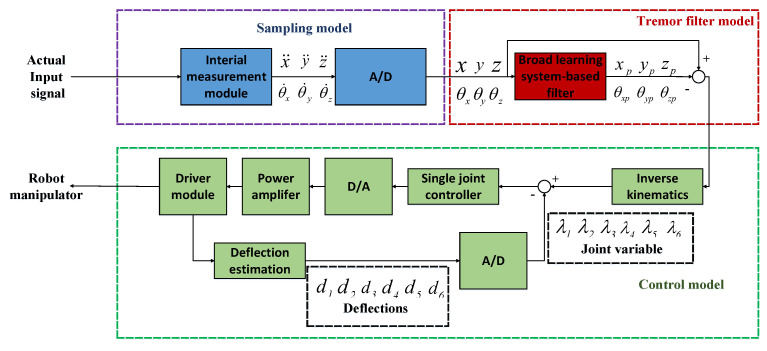
Block diagram of the broad-learning-system-based tremor filter.

By incorporating the BLS network algorithm, the proposed BLSF effectively forecasts tremor signals, as depicted in Figure 6. The three-axis compensation signals, namely, $x_{p}$, $y_{p}$, and $z_{p}$, and $\theta_{xp}$, $\theta_{yp}$, and $\theta_{zp}$, exhibit equal magnitudes but opposite phases in comparison to the tremor signals. This unique characteristic allows them to effectively neutralize the tremor signals present in the actual signals *x*, *y*, and *z*.

## 5. Simulation Experiments

A mainstream SVM algorithm in the field of machine learning was used. Its model was built based on solving convex optimization problems in optimization problems. At the same time, kernel functions were used to replace the nonlinear mapping of high-dimensional space to realize the role of processing high-dimensional space data in the low-dimensional calculation. The desired classification flat was only related to the support vector samples, thereby enabling the SVM-based algorithm to have the ability of small sample learning. However, this algorithm suffers from poor performance in canceling tremors because of their characteristics.

Hence, in this subsection, we compared the broad-learning-system-based algorithm model with the support vector machine algorithm for the tele-operation systems, which were mainly based on the MATLAB Robotics and Libsvm toolbox.

### 5.1. Model Evaluation Metrics

Determing whether a model has the ability of classification or regression can be judged by some evaluation metrics, such as the Euclidean distance error. In this topic, the (1) sum square error (*SSE*); (2) root mean square error (*RMSE*); and (3) regression determination coefficient ($R^{2}$) were used as the evaluation strategies of the various network models.

(37) $$SSE=\sum_{t=1}^{T}(n_{ref}(t{)-n_{pre}\left(t\right))}^{2}$$

(38) $$RMSE=\sqrt{\frac{\sum_{t=1}^{T}(n_{ref}(t{)-n_{pre}\left(t\right))}^{2}}{N}}$$

(39) $$R^{2}=1-\frac{\sum_{t=1}^{T}n_{ref}\left(t\right)-n_{pre}\left(t\right)}{\sum_{t=1}^{T}n_{ref}\left(t\right)-{\bar{n}}_{ref}\left(t\right)},$$

where *T* represents the periods, and *N* is the number of samples. ${\bar{n}}_{ref}\left(t\right)$ is the mean values of $n_{ref}$.

### 5.2. Data Pre-Processing

In order to satisfy the same distribution of the input data and to prevent the difference of varying data from being large, we pre-processed the input data. Specifically, we used z-score normalization processing, which allows the input data to be adjusted to present a standard normal distribution, i.e., the Gaussian distribution, which satisfies the zero mean and the one variance.

(40) $$s=\frac{s_{i}-min(s)}{max(s)-min(s)},$$

where *s* represents the input vectors on the three-axis directions in the original input data. After standardization, all the elements in the vector are normalized to [0, 1], which can accelerate the training and learning speed of the network.

### 5.3. Parameter Settings

The simulation experiment had a sampling time of 200 s with an interval time of 1 s, thus resulting in a total of 200 sampling points. In MATLAB, we built a simulation robot, and its joint parameters are given in Table 1. Additionally, other simulation parameters, such as network nodes and activation functions, were set according to the following specifications.

Noise signals were used to simulate the tremor signals of the human hand. We added these simulated signals into the trajectory, and they were set as two parts: (1) the low-frequency part and (2) the high-frequency part. The physiological tremor signal is defined as the joint angle signal of the haptic device that comes into contact with the human hand. Assuming the absence of any accompanying physiological tremor in the human body, the joint angle information of the joystick is denoted by $q_{d}$. However, after being affected by tremors, the joint angle is updated as the following:

(41) $$\begin{array}{c} q\left(t\right)=q_{d}\left(t\right)+n\left(t\right). \end{array}$$

**Figure 7:**
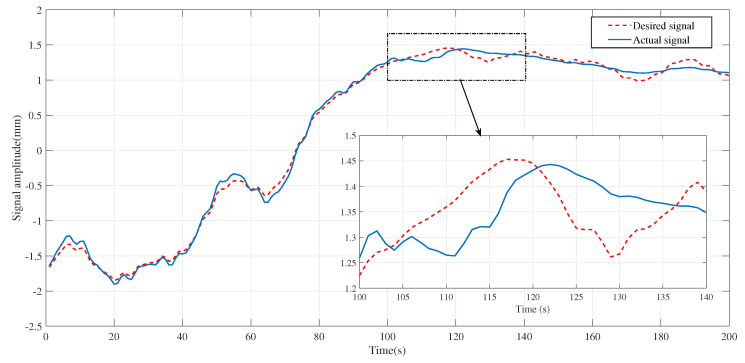
Expected values and actual values when the operator was operating.

In Figure 7, the presence of a physiological tremor can cause the operating human hand to deflect during operations, particularly during slow movements. This amplifies the effect of the physiological tremor, and the subplot in Figure 7 demonstrates that the operator’s actual trajectory deviated significantly from the desired target trajectory.

For the BLS-based tremor filter, the sparse regularization parameter *C* was set to $2^{-30}$, while the reduction parameter *s* for the enhanced node was set to 0.8. The broad learning system consists of N11=10 feature nodes, N2=80 feature node windows, and N33=200 enhanced nodes for each window. The number of added feature nodes was m1=10, the number of enhancement nodes related to the incremental feature nodes per increment step was m2=20, and the number of enhancement nodes in each incremental learning was m3=50. The activation function ξ(·)=tanh(·) was used for mapping the feature nodes to the enhanced nodes. For the SVM-based filter, a method based on epsilon support vector regression was used with a loss function parameter *p* set to 0.4, which indicated the penalty degree for the input data. Moreover, the radial basis function (RBF) was selected as the kernel function of the network.

## 6. Tremor Forecast Results

**Figure 8:**
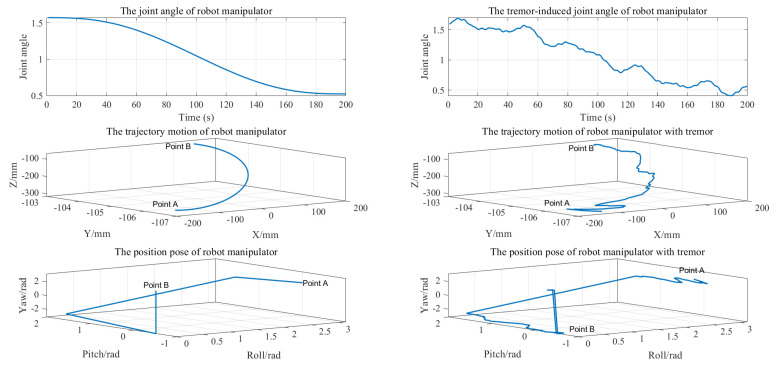
Joint angle and motion trajectory of robot manipulator with tremors and without tremors.

In this section, the comparison simulation experiments w.r.t. the broad learning system and support vector machine were achieved under the simulated physiological tremor signal. As shown in Figure 8, the simulated manipulator was built in our MATLAB platform, and the motion trajectory and the joint angle of the manipulator with tremors and without tremors are given. In millimeters, we can see that the joint angle and the motion trajectory with tremors deviated from the desired trajectory.

**Figure 9:**
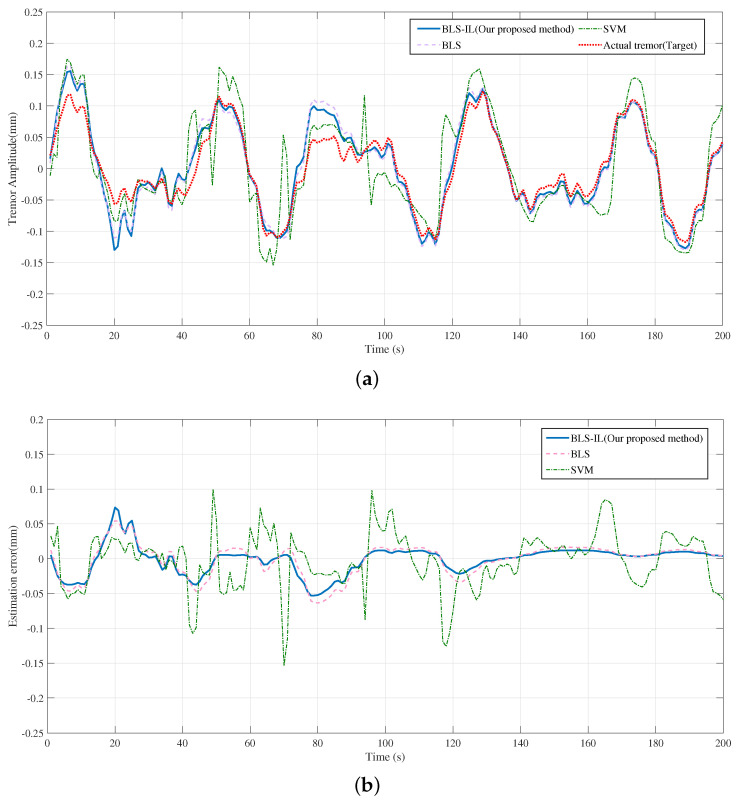
The results of canceling tremors based on different approaches. (**a**) In the case of tremors, prediction values based on different approaches. (**b**) In the case of tremors, the error between tremor values and prediction values based on different approaches.

**Figure 10:**
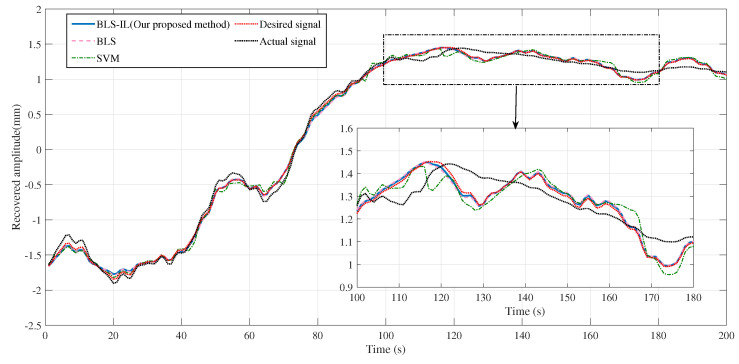
Tremor attenuation performance based on different approaches.

**Figure 11:**
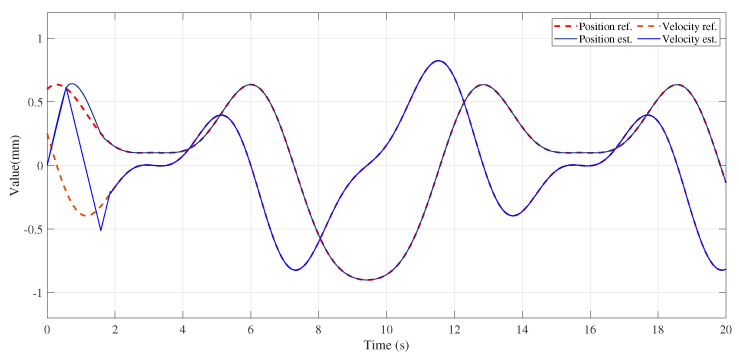
Position and velocity control resulting from applying sliding mode controller.

Figure 9a shows the prediction and estimation ability for the broad learning system and the support vector machine, where four curves are shown in the figure, which are the broad learning system, the incremental broad learning system, the support vector machine, and the actual tremor-induced offset. Since the ability of the broad learning system to predict the tremor-induced offset trajectory reached a saturation state, there was still no obvious effect improvement after the reinforcement of incremental learning, which indicates that the ability of the broad learning system to learn time sequence signals is limited to some extent. To compare the performance of different algorithms, we can observe the error curve shown in Figure 9b. As we can see, the SVM error fluctuated, whereas the BLS error decreased gradually over time. In summary, the proposed BLS algorithm outperformed the SVM. This conclusion is supported by the evaluation metrics in Table 2, which indicate that a good regression model should have a high determination coefficient $R^{2}$. In Figure 10, we can observe the recovery trajectory of a tele-operation system under the influence of various filters. By comparing the recovery trajectories across multiple filters, we can gain insights into the relative effectiveness and limitations of each filter in achieving the desired outcome. As shown in Figure 11, the position and velocity control were achieved by applying a sliding mode controller. First, the position and velocity references were input into the controller, which generated a control signal that was then applied to the system. The sliding mode controller ensures that the system tracks the desired position trajectory while also regulating the velocity. The resulting system behavior is shown in the position and velocity plots.

Overall, the teleoperation robot system applied the sliding mode controller to obtain a good performance, while our proposed filter was efficient in canceling tremors.

## 7. Conclusions

In this paper, we proposed a simple and efficient network model, the incremental broad learning system, as a tremor filter architecture for the current application issues of deep learning and machine learning in tele-operation. Unlike deep learning algorithms that have many hyperparameters, our proposed approach simplifies the learning process and avoids such complexities. Furthermore, the support vector machine often suffers from poor precision in regression tasks, and our novel architecture was designed to overcome this issue. We combined it with incremental learning algorithms to rapidly improve performance. Additionally, our proposed sliding mode controller provided greater stability and faster response performance when compared to traditional controllers. The simulation results and performance metrics demonstrated the effectiveness of our approach in attenuating tremors.

In future work, we will delve deeper into the feature extraction module of the broad learning system to improve its ability to eliminate physiological tremors to the best of our ability. Although our experimental results demonstrated the efficient elimination of physiological tremors by the broad-learning-system-based, we believe that there is still room for improvement.

## Figures and Tables

**Table 1:** The MATLAB-based simulation robot arm joint parameters.

i	Theta	d	a	Alpha	Offset
1	q1	105	0	π/2	0
2	q2	0	−174	0	−π/2
3	q3	0	−174	0	0
4	q4	76	0	π/2	−π/2
5	q5	80	0	−π/2	0
6	q6	44	0	0	0

**Table 2:** Canceling tremor results of different filters.

Different Methods and Metrics	*SSE*	*RMSE*	$R^{2}$	Train Time
Broad learning system filter	0.0687	0.0026	80.06%	0.118
Incremental broad learning system filter	0.0587	0.0024	82.94%	0.122
Support vector machine filter	0.0918	0.0303	73.35%	0.278

## Data Availability

Not applicable.
